# EVG-Based Periarterial/Perivenous Invasion (periA/V) as a High-Sensitivity Surrogate Marker for Lymph Node Metastasis in pT1 Invasive Breast Carcinoma of No Special Type

**DOI:** 10.3390/diseases14050177

**Published:** 2026-05-19

**Authors:** Chikara Mashiba, Akihiro Shioya, Takanobu Takata, Motona Kumagai, Miyako Shimasaki, Takeru Oyama, Yusuke Haba, Emi Morioka, Masafumi Inokuchi, Sohsuke Yamada

**Affiliations:** 1 Department of Pathology and Laboratory Medicine, Kanazawa Medical University, 1-1 Daigaku, Uchinada, Kahoku 920-0293, Ishikawa, Japan; cmassy@kanazawa-med.ac.jp (C.M.); oym@kanazawa-med.ac.jp (T.O.); sohsuke@kanazawa-med.ac.jp (S.Y.); 2 Division of Molecular and Genetic Biology, Department of Life Science, Medical Research Institute, Kanazawa Medical University, 1-1 Daigaku, Uchinada, Kahoku 920-0293, Ishikawa, Japan; takajjjj@kanazawa-med.ac.jp; 3 Department of Pharmacy, Kanazawa Medical University Hospital, 1-1 Daigaku, Uchinada, Kahoku 920-0293, Ishikawa, Japan; 4 Department of Pathophysiological and Experimental Pathology, Kanazawa Medical University, 1-1 Daigaku, Uchinada, Kahoku 920-0293, Ishikawa, Japan; kumagaim@kanazawa-med.ac.jp (M.K.); miya0807@kanazawa-med.ac.jp (M.S.); 5 Department of Breast Oncology, Kanazawa Medical University, 1-1 Daigaku, Uchinada, Kahoku 920-0293, Ishikawa, Japan; haba@kanazawa-med.ac.jp (Y.H.); emi-mori@kanazawa-med.ac.jp (E.M.); inokuchi@kanazawa-med.ac.jp (M.I.)

**Keywords:** breast cancer, lymph node metastasis, periarterial or perivenous invasion, periA/V, perivascular invasion, Elastica–van Gieson staining, pT1, histological surrogate marker

## Abstract

Background/Objectives: Conventional assessments of lymphatic invasion in the primary tumor may fail to identify lymph node metastasis (LNM) in breast cancer. We evaluated periarterial or perivenous invasion (periA/V), using Elastica–van Gieson (EVG)-stained sections, as a histological marker associated with LNM in invasive breast carcinoma of no special type (IBC-NST), focusing on the impact of invasive tumor size. Methods: We retrospectively analyzed 213 IBC-NST cases. PeriA/V was defined as tumor nests in direct contact with perivascular elastic fibers on EVG-stained sections. Diagnostic performance was compared with that of conventional LI markers (hematoxylin and eosin and D2-40), with stratified analyses by pathological T category (pT1 vs. pT2–4) and pT1 subcategories (pT1a, pT1b, and pT1c). Results: LNM was observed in 87 cases (40.8%). Overall, periA/V demonstrated high sensitivity (97.7%) and negative predictive value (NPV; 93.5%). In pT1 tumors (*n* = 130), periA/V achieved 100% sensitivity and 100% NPV (27/27), and was consistently present in all node-positive pT1b–c tumors. In multivariate analyses, periA/V remained independently associated with LNM in the pT1 group (odds ratio [OR]: 16.08, *p* = 0.003) and pT1c subgroup (OR: 14.7, *p* = 0.010). In pT2–4 tumors, periA/V became frequent regardless of nodal status, indicating reduced discriminatory value. Conclusions: In this exploratory single-center cohort, EVG-based periA/V demonstrated high sensitivity for LNM in pT1 IBC-NSTs, with periA/V negativity consistently observed among node-negative cases. These preliminary findings suggest that periA/V may potentially contribute to LNM risk assessment in early-stage breast cancer.

## 1. Introduction

In breast cancer pathology, 45–78% of Lymph node metastasis (LNM)-positive cases reportedly lack detectable lymphatic invasion (LI) in the primary tumor [1,2,3,4,5,6,7,8,9,10]. As LNM is generally considered to arise via lymphatic pathways [11,12,13], LI would be expected to be detected at least as frequently as LNM. However, this expectation is often not met in routine histopathology, suggesting a discrepancy between the biological mechanisms of tumor spread and current histological assessment methods.

In daily practice, LI is identified by confirming the presence of tumor cells within lymphatic spaces on hematoxylin and eosin (HE)-stained sections and/or by immunohistochemistry for podoplanin (D2-40), a lymphatic endothelial marker [14,15]. Nevertheless, the frequent occurrence of LNM without detectable LI suggests that these conventional approaches lack sufficient sensitivity. Furthermore, D2-40 can stain mammary myoepithelium and Schwann cells and is therefore not perfectly specific for the lymphatic endothelium [16,17,18,19]. Such non-specific staining can lead to misinterpretation of LI, representing a diagnostic pitfall in risk assessment.

To address this issue, we focused on perivascular cell invasion. Using Elastica–van Gieson (EVG)-stained elastic fibers as histological landmarks, this approach evaluates tumor nests in contact with perivascular regions. Periarterial (periA) perivenous (periV) invasions were defined as contact around arteries and veins, respectively; cases exhibiting either feature were classified as periarterial or perivenous invasion (periA/V)-positive [1]. In our previous study, periA/V was strongly associated with LNM, demonstrating high sensitivity (95.5%), high negative predictive value (NPV; 88.2%), and low negative likelihood ratio (0.19). As lymphatic vessels are anatomically distributed in close proximity to blood vessels within mammary tissues, perivascular tumor contact may serve as a sensitive surrogate marker for lymphatic network proximity. Moreover, EVG staining provides clear visualization of elastic fibers as histological landmarks, enabling objective and reproducible assessment.

In our previous analysis, invasive tumor size was significantly associated with LNM using a 20-mm cutoff, consistent with its established prognostic impact. However, the extent to which the diagnostic value of periA/V varies across tumor size categories remains incompletely elucidated. Therefore, in this study, we increased the sample size and performed detailed stratification according to invasive tumor size to investigate the relationship between periA/V and LNM across categories. We also compared periA/V with conventional LI indicators to clarify the impact of tumor size on their diagnostic performance.

## 2. Materials and Methods

### 2.1. Case Selection

Of the 452 patients who underwent surgical resection for breast cancer at Kanazawa Medical University Hospital between January 2018 and December 2023, 213 cases of invasive breast carcinoma of no special type (IBC-NST) were analyzed. To minimize clinicopathological heterogeneity and the effects of preoperative treatment, the cohort was restricted to surgically resected IBC-NST cases without neoadjuvant therapy. The exclusion criteria were special histological subtypes (including invasive lobular or apocrine carcinoma); ductal carcinoma in situ (DCIS); microinvasive carcinoma; male breast cancer; recurrent disease; and multicentric or bilateral breast cancer, defined as distinct, separate primary tumors. In contrast, multifocal invasive foci presumed to arise from the same primary lesion were evaluated as clinicopathological variables.

### 2.2. Clinical and Pathological Data Collection

Clinical information for the included patients was retrieved from electronic medical records. Pathological data were obtained from original diagnostic reports based on surgical specimens, and slides were re-evaluated when necessary. Invasive tumor size was categorized according to the TNM classification framework, given its established prognostic significance. In this study, tumors were primarily stratified by the maximum invasive diameter into pT1 (≤20 mm) and pT2–4 (>20 mm) categories. Furthermore, to investigate the diagnostic performance of periA/V across different stages of early tumor progression, pT1 cases were further subdivided into three subcategories: pT1a (≤5 mm), pT1b (6–10 mm), and pT1c (11–20 mm). Although the T1, T2, and T3 categories are defined by tumor size, a small number of T4 cases in our cohort were also included in the analysis. Histological review and identification of findings were performed collaboratively by the first author (CM) and three pathologists (A.S., M.K., and S.Y.), and all findings were finalized through a consensus discussion. In this study, LNM was defined as the histopathological presence of tumor cells in lymph nodes. Patients were divided into two groups: N0 and N+ (including N0[i+], N1mi, and N1–3). This grouping was adopted because our primary focus was on the pathological correlation between periA/V status and microscopic tumor dissemination to lymph nodes, irrespective of clinical staging implications.

### 2.3. Preparation of Specimens

A representative section from each resected tumor was selected for pathological evaluation. Serial 4-μm sections were prepared from the corresponding formalin-fixed, paraffin-embedded tissue blocks. The sections were subjected to HE, EVG, and immunohistochemical staining for D2-40 and p63. Immunostaining for the estrogen receptor (ER), progesterone receptor (PgR), human epidermal growth factor receptor 2 (HER2), and Ki-67 was performed as part of a routine diagnostic workup. HE staining was performed using Tissue-Tek Prisma Plus (Sakura Finetek, Tokyo, Japan) with the manufacturer-recommended reagents (hematoxylin 3G for 5 min and eosin for 1.5 min). EVG staining was performed manually. After deparaffinization, sections were stained with Weigert’s resorcin–fuchsin solution for at least 3 h, briefly differentiated in 100% ethanol three times, rinsed under running water, stained with Weigert’s iron hematoxylin for 5 min, washed again, and counterstained with van Gieson solution for 7 min, followed by differentiation in 70% alcohol for a few seconds three times, dehydration, clearing in xylene, and coverslipping. Immunohistochemistry (IHC) was performed using an automated system. D2-40 staining was performed on a Bond-Max autostainer (Leica Biosystems, Nussloch, Germany) using the Bond Polymer Refine Detection kit, including heat-induced epitope retrieval in Bond Epitope Retrieval Solution 2 (pH 9.0) for 20 min. ER, PgR, HER2, Ki-67, and p63 staining were performed using the VENTANA BenchMark ULTRA platform (Roche Diagnostics, Tucson, AZ, USA) according to the manufacturer’s protocols. Ki-67 and p63 staining was conducted with CC1 mild conditioning and 32-min incubation with primary antibodies. The following primary antibodies were used: D2-40 (mouse monoclonal, 1:5; Nichirei), p63 (4A4, ready-to-use; Nichirei), ER (SP1, ready-to-use; Roche), PgR (1E2, ready-to-use; Roche), HER2 (4B5, ready-to-use; Roche), and Ki-67 (MIB-1, 1:50; DAKO).

### 2.4. Definition of Pathological Parameters

The pathological variables analyzed in this study were categorized as conventional diagnostic indicators and specific parameters of perivascular involvement. LI, venous invasion (VI), and perineural invasion (PNI) were evaluated according to standard histopathological criteria [14,15,20,21]. Retraction artifacts were identified based on established morphological descriptions reported in the literature [22,23]. Conversely, parameters focusing on perivascular and periductal involvement—periA, periV, periA/V, and periductal invasion (periD)—were defined and assessed based on the criteria established in our previous study [1]. The specific evaluation criteria for each parameter are detailed below.

### 2.5. Lymphatic Invasion

On HE-stained sections, LI was diagnosed when tumor cells were observed within the space lined by endothelial cells. On D2-40 immunostaining, LI was defined as the presence of tumor cells within the D2-40–positive lymphatic lumen.

### 2.6. Venous Invasion

On HE- or EVG-stained sections, VI was defined as the presence of tumor cells within a vessel judged to be a vein by the evaluating pathologist.

### 2.7. Perivascular and Periductal Invasion

**Figure 1:**
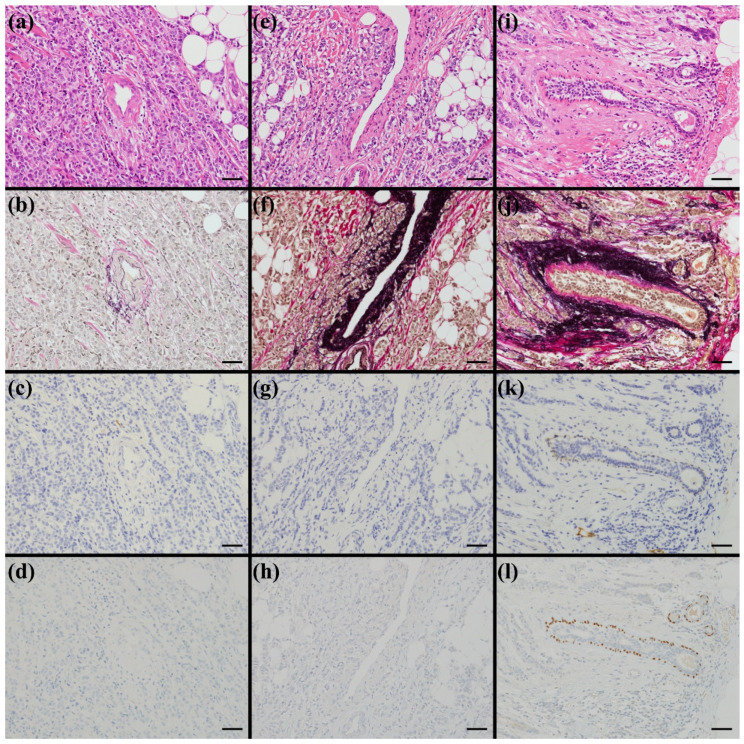
Histological definitions of periA, periV, and periD. Panels (**a**–**d**) illustrate periA, (**e**–**h**) periV, and (**i**–**l**) periD. For each set, (**a**,**e**,**i**) are HE-stained sections, (**b**,**f**,**j**) EVG-stained sections, (**c**,**g**,**k**) D2-40 immunostaining, and (**d**,**h**,**l**) p63 immunostaining. Arteries, veins, and mammary ducts were identified primarily on EVG sections. In EVG-stained sections, elastic fibers are sharply highlighted in dark purple to black. Tumor nests directly contacting elastic fibers around these structures were defined as periA, periV, and periD, respectively. For practical vessel classification, vessels with an identifiable internal elastic lamina were regarded as arteries, whereas those without were regarded as veins. periA/V was defined when either periA or periV was present. Serial sections (HE, EVG, D2-40, and p63) were reviewed to confirm histological correspondence across stains; final judgments were made using the stain specified in each definition. All images were acquired at ×200 magnification (scale bar, 50 μm). periA, periarterial invasion; periV, perivenous invasion; periD, periductal invasion; HE, hematoxylin and eosin; EVG, Elastica–van Gieson.

HE- and EVG-stained slides were reviewed to classify arteries, veins, and mammary ducts. Arteries and veins were distinguished based on the presence of an internal elastic lamina on EVG staining. Vessels with a clearly identifiable elastic lamina were regarded as arteries, whereas those without were regarded as veins. Although small arteries occasionally lack a distinct elastic lamina, this simplified criterion was adopted for practical consistency. To differentiate mammary ducts from blood vessels, the epithelial architecture was used as the primary feature. Structures with a bilayered epithelium composed of luminal epithelial cells and an outer myoepithelial layer were classified as mammary ducts. When the distinction was uncertain, the presence of myoepithelial cells was confirmed by p63 immunostaining. Based on these structural criteria, periA, periV, and periD were defined as tumor nests in direct contact with elastic fibers surrounding arteries, veins, and mammary ducts, respectively (Figure 1). To evaluate perivascular invasion as a single composite parameter, patients with either periA or periV were categorized as periA/V.

**Figure 2:**
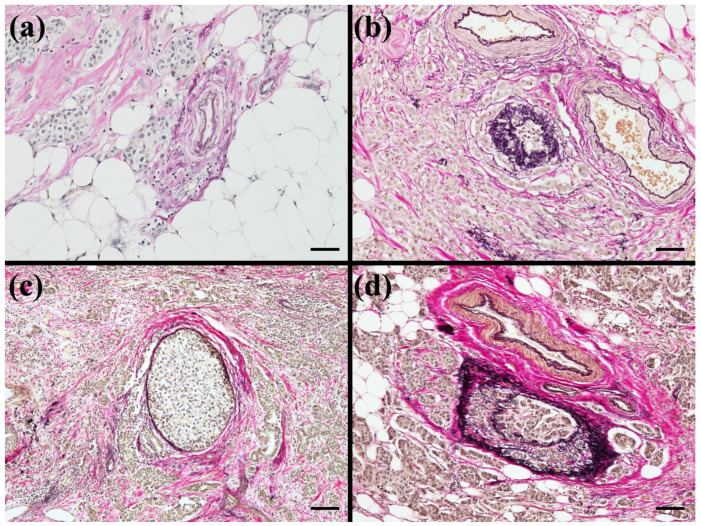
Supplementary assessment criteria. In EVG-stained sections, elastic fibers are sharply highlighted in dark purple to black. (**a**) Example of focal/limited tumor contact with perivascular elastic fibers. (**b**) Example of extensive/circumferential tumor contact with perivascular elastic fibers. In both (**a**,**b**), any degree of contact was regarded as positive (qualitative assessment). Images: ×200 (scale bar, 50 μm). (**c**) Invasive tumor nests contacting elastic fibers around a mammary duct adjacent to ductal carcinoma in situ (DCIS). (**d**) Invasive carcinoma contacting/infiltrating elastic fibers around a vessel with venous invasion. In (**c**,**d**), intraluminal findings (empty vs. tumor-filled) did not affect the evaluation; positivity was defined by direct contact with external elastic fibers. Images: ×100 (scale bar, 100 μm).

To ensure a consistent and reproducible evaluation of periA, periV, periA/V, and periD, the following four considerations were systematically applied:

(1) Structural Identification: To ensure accurate identification of lymphatic vessels, blood vessels, and mammary ducts, up to four serial sections (HE, EVG, D2-40, and p63) were reviewed when necessary. However, the final determinations were made solely based on the stain specified in each definition (such as EVG for periA/V/D; Figure 1).

(2) Qualitative Assessment: The extent of contact between the tumor nest and the surrounding elastic fibers was assessed qualitatively rather than quantitatively. Any degree of contact, slight/focal or circumferential, was regarded as a positive finding (Figure 2a,b).

(3) Location Independence: Findings of periA, periV, periA/V, or periD were recorded regardless of their location within the specimen, including both the invasive front and the tumor center.

(4) Independence from Intraluminal Findings: The status within the lumen, whether empty or occupied by tumor cells (for example, DCIS or VI), did not influence the evaluation. The assessment focused exclusively on the outer boundary. A finding was defined as positive when an invasive tumor nest was in direct contact with the external elastic fibers, regardless of the findings inside the vessel or duct (Figure 2c,d).

These criteria were intentionally set with the same design rationale in mind, prioritizing detectability over restrictive specificity.

### 2.8. Retraction Artifact and Perineural Invasion

Retraction artifacts were evaluated on HE-stained sections and defined as clear stromal spaces or clefts appearing to separate tumor nests from the surrounding stroma. Although these artifacts are relatively common in breast cancer, they can mimic true lymphatic spaces and reportedly correlate with LI and LNM [22,23]. PNI was similarly assessed on HE-stained sections and defined according to standard criteria [20,21]. Given that both retraction artifacts and PNI are histological findings that can be assessed during routine pathological examinations, they were included as clinicopathological variables in the multivariate analysis to evaluate their relationship with LNM.

### 2.9. Pathological Evaluation and Consensus

Initially, a graduate student (C.M.) and a pathologist (A.S.) jointly reviewed all sections to identify and mark the sites where pathological findings were observed. Subsequently, four investigators (C.M., A.S., M.K., and S.Y.) simultaneously examined the marked sites using a multiheaded microscope. Each finding was discussed in detail to ensure that it met the predefined criteria, and a final consensus was reached regarding its positivity or negativity. The use of elastic fibers as stable histological landmarks on EVG-stained sections was considered to enhance the objectivity and reproducibility of the periA/V assessment compared with conventional LI evaluation.

### 2.10. Statistical Analyses

The relationship between each pathological finding and LNM was evaluated using Fisher’s exact test, contingency-table-based diagnostic performance indices, and univariate and multivariate logistic regression analyses. Multivariate logistic regression analysis was performed to compare the independent predictive value of perivascular invasion parameters (periA, periV, and periA/V) against conventional LI markers (LI on HE and LI on D2-40) and other histological findings potentially related to lymphatic invasion, including retraction artifacts and perineural invasion, to identify the most informative surrogate for LNM among these parameters. Standard analyses were performed using EZR (version 1.68; Saitama Medical Center, Jichi Medical University, Saitama, Japan), a graphical user interface for the R statistical environment (version 4.3.1; R Foundation for Statistical Computing, Vienna, Austria) [24]. When complete or quasi-complete separation was encountered, specifically when the maximum likelihood estimation failed to yield finite parameter estimates, logistic regression with Firth’s bias reduction method was conducted. This correction was applied solely to stabilize estimation, and no variable selection procedure was used. All statistical tests were two-tailed, and a *p*-value < 0.05 was considered statistically significant. To further assess the independent predictive value of periA/V beyond the primary analysis, after adjusting for key clinicopathological variables, an additional multivariate logistic regression analysis was performed. This analysis incorporated tumor size (>20 mm vs. ≤20 mm), nuclear grade (1 vs. 2–3), histological grade (1 vs. 2–3), multifocality, LI on HE, and LI on D2-40 as covariates.

## 3. Results

### 3.1. Patient Characteristics

**Figure 3:**
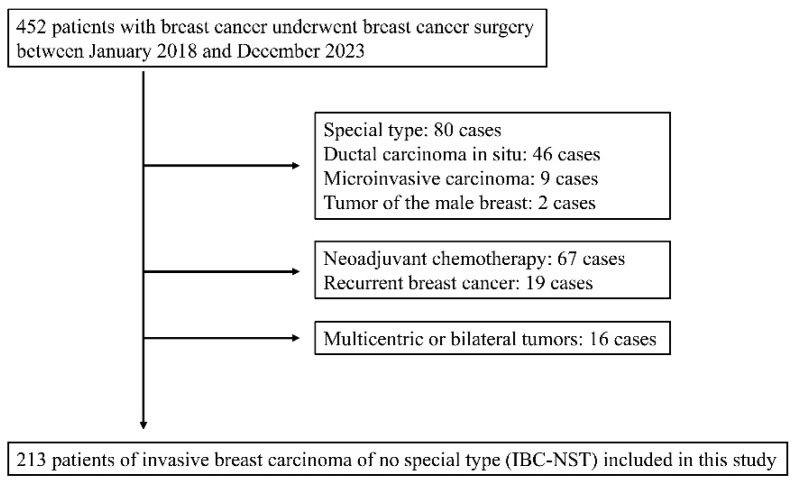
Flowchart of patient selection. A total of 452 patients with breast cancer underwent surgery between January 2018 and December 2023. The following cases were excluded: special types of breast cancer (*n* = 80), ductal carcinoma in situ (*n* = 46), microinvasive carcinoma (*n* = 9), and male breast tumors (*n* = 2). In addition, patients who received neoadjuvant chemotherapy (*n* = 67), those with recurrent breast cancer (*n* = 19), and those with multicentric or bilateral tumors (*n* = 16) were excluded. Ultimately, 213 patients with invasive breast carcinoma of no special type (IBC-NST) were included in this study.

After applying the inclusion and exclusion criteria (Figure 3), 213 patients with IBC-NST were included. Clinicopathological characteristics are summarized in Table 1. The mean and median ages were 60.7 and 61 years, respectively (range, 23–92 years). Seventy-two patients were premenopausal, and 141 were postmenopausal. Regarding the surgical procedures, 107 patients underwent mastectomy (including skin- and nipple-sparing mastectomy), 106 underwent partial mastectomy, and 52 underwent axillary lymph node dissection. Tumors were located almost equally in the left (*n* = 106) and right (*n* = 107) breasts. The upper outer quadrant was the most common anatomical site (*n* = 90). Nuclear grades 1, 2, and 3 were observed in 87, 37, and 89 patients, respectively, whereas histological grades I, II, and III were observed in 73, 84, and 56 patients, respectively. Unifocal tumors were present in 140 patients, whereas 73 had multifocal tumors. The mean and median invasive tumor sizes were 19.8 and 17 mm, respectively (range, 2–90 mm). Pathological T categories were distributed as follows: pT1 (≤20 mm), 130 cases (11 pT1a [≤5 mm], 44 pT1b [6–10 mm], and 75 pT1c [11–20 mm]); pT2 (21–50 mm), 73; pT3 (>50 mm), 6; and pT4, 4. Regarding axillary lymph node status, 126 patients were node-negative (N0), while 87 were node-positive (N+), including 7 N0(i+), 14 N1mi, 46 N1, 14 N2, and 6 N3. Distant metastases at diagnosis were present in two patients. Pathological TNM stages were as follows: IA, 104 cases; IB, 5; IIA, 45; IIB, 32; IIIA, 17; IIIB, 3; IIIC, 5; and IV, 2. Hormone receptor status was evaluated using the J-score system [25].

For ER, J-scores of 3b, 3a, 2, 1, and 0 were observed in 193, 6, 1, 2, and 11 cases, respectively. For PgR, J-scores of 3b, 3a, 2, 1, and 0 were observed in 130, 51, 10, 1, and 21 cases, respectively. HER2 expression was evaluated using IHC according to the ASCO/CAP method [26]. Regarding HER2, IHC 3+ was identified in 10 cases, IHC 2+ with FISH positivity was observed in 8 cases, and IHC 2+ with FISH negativity, IHC 1+, and IHC 0 were observed in 27, 130, and 38 cases, respectively. The Ki-67 labeling index (hotspot method) showed a mean of 25.9% and a median of 21.1% (range, 0.5–88.6%), with 113 tumors (53.1%) exhibiting a labeling index >20%.

### 3.2. Correlation Between LNM and Clinicopathological Characteristics

The 213 patients with IBC-NST were categorized into two groups based on LNM status: N0 group (*n* = 126) and N+ group (including N0(i+) and N1–3; *n* = 87). The clinicopathological characteristics of the patients are summarized in Table 2. Regarding patient demographics, no significant differences were observed between the N0 and N+ groups in terms of menopausal status (*p* = 0.306), tumor laterality (*p* = 0.404), or anatomical subsite (*p* = 0.417). Conversely, several tumor-related factors were significantly associated with LNM. Using a cutoff of 20 mm, invasive tumors >20 mm were significantly more frequent in the N+ group than in the N0 group (64.4% vs. 22.2%, *p* < 0.001). Higher tumor grades were also strongly associated with LNM (*p* < 0.001); specifically, Nuclear Grade 3 was observed in 57.5% of the N+ group compared with 31.0% of the N0 group. Similarly, Histological Grade III was more frequent in the N+ group (35.6%) than in the N0 group (19.8%; *p* < 0.001). Furthermore, multifocal tumors were significantly more common in the N+ group (46.0%) than in the N0 group (26.2%; *p* = 0.003). The diagnostic performance of various morphological findings for identifying LNM was evaluated, and the results are summarized in Table 3. Notably, periA/V was identified in 85 of 87 cases (97.7%) in the N+ group. In contingency-table analysis, periA/V demonstrated a high sensitivity of 0.977 and a negative predictive value (NPV) of 0.935, with a negative likelihood ratio (LR–) of 0.10. Distinct diagnostic characteristics were observed when periA/V was compared with conventional pathological indicators. LI sensitivity was markedly lower than that of periA/V: 0.333 on HE-stained sections and 0.356 on D2-40 immunostaining. In contrast, LI showed high specificity (0.841 on HE and 0.817 on D2-40) but low sensitivity, indicating a different diagnostic profile from that of periA/V in this cohort. VI in EVG-stained sections was significantly associated with LNM (*p* < 0.001), with a sensitivity of 0.759, which did not reach that of periA/V (0.977). Retraction artifacts were identified in 58.6% of the patients in the N+ group (*p* < 0.001). In contrast, periD and PNI were not significantly associated with LNM (both *p* = 0.062).

### 3.3. Comparative Analysis of Histological Surrogates Associated with LNM

We performed logistic regression analyses to identify the morphological findings most robustly associated with LNM (Table 4). The objective was to directly compare the strength of the association between various histological features and LNM, rather than to examine clinical risk factors. In the univariate analysis, all histological indicators, except periD (*p* = 0.0525), were significantly associated with LNM. Notably, perivascular invasion-related features had remarkably high odds ratios (ORs). periA/V exhibited the highest OR of 12.70 (95% CI: 2.94–54.80), followed by periV with an OR of 8.30 (95% CI: 2.83–24.30). Conventional indicators, such as lymphatic invasion (OR 2.65 for HE; OR 2.48 for D2-40), retraction artifact (OR 2.64, *p* = 0.001), and PNI (OR 1.85, *p* = 0.046), also showed significant associations; however, their ORs were considerably lower than those of periA/V and periV. To determine the independent associations between these surrogates, a multivariate analysis was conducted. periD, which was not significant in univariate analysis, was excluded. Furthermore, to avoid multicollinearity and specifically test whether the integrated assessment of perivascular invasion (periA/V) provided incremental information beyond conventional evaluations, periA/V was selected as the representative variable, whereas individual periA and periV were excluded from the model. The multivariate analysis revealed that only periA/V (OR 9.69, 95% CI: 2.16–43.50) and retraction artifact (OR 2.41, 95% CI: 1.30–4.48) remained independently associated with LNM. Conversely, conventional lymphatic invasion (HE and D2-40) and PNI were not significant in the presence of periA/V. The variance inflation factor (VIF) for each variable ranged from 1.03 to 1.54, indicating negligible multicollinearity. These results suggest the association with LNM was stronger for periA/V than that for conventional lymphatic invasion markers in this cohort.

### 3.4. Multivariate Analysis Adjusting for Clinicopathological Variables

To further assess whether periA/V retains independent predictive value after adjusting for key clinicopathological variables, a multivariate logistic regression analysis was conducted. This analysis incorporated nuclear grade, histological grade, multifocality, invasive tumor size, LI on HE, and LI on D2-40 as covariates. In the univariate analysis, all variables were significantly associated with LNM. In the multivariate model, periA/V remained an independent predictor of LNM (OR 12.400, 95% CI 2.590–59.70, *p* = 0.002), as did invasive tumor size (OR 3.730, 95% CI 1.860–7.460, *p* < 0.001) and multifocality (OR 2.880, 95% CI 1.420–5.850, *p* = 0.004). In contrast, nuclear grade, histological grade, LI on HE, and LI on D2-40 were not independently associated with LNM in the multivariate model. The results of univariate and multivariate analyses are detailed in Table 5.

### 3.5. Stratified Analysis by Tumor Size

To assess whether tumor size modified the association between each indicator and LNM, cases were stratified into pT1 (≤20 mm; *n* = 130) and pT2–4 (>20 mm; *n* = 83) groups (Table 6). In the pT1 group, periA/V was present in 100.0% (31/31) of the node-positive cases and 72.7% (72/99) of the node-negative cases (*p* < 0.001). PeriA/V negativity was observed only in node-negative cases (NPV 100.0%, 27/27). Conventional lymphatic invasion (LI) showed significantly lower positivity rates (HE: 35.5% [11/31] vs. 11.1% [11/99], *p* = 0.004; D2-40: 32.3% [10/31] vs. 14.1% [14/99], *p* = 0.033) and low sensitivity (32.3–35.5%) despite its high specificity (85.9–88.9%). Retraction artifacts (*p* = 0.513) and PNI (*p* = 0.086) were not significantly associated with nodal status. In the pT2–4 group, periA/V was frequently observed regardless of nodal status (96.4% [54/56] in node-positive vs. 92.6% [25/27] in node-negative cases; *p* = 0.593). No significant associations with nodal status were observed for LI on HE (*p* = 1.000), LI on D2-40 (*p* = 0.810), retraction artifacts (*p* = 0.089), or PNI (*p* = 1.000), suggesting a reduced discriminatory power of these indicators in larger tumors.

### 3.6. Stratified Logistic Regression Analysis by Tumor Size

To further evaluate the influence of tumor size on the association between each histological indicator and LNM, logistic regression analyses were performed separately for the pT1 (≤20 mm) and pT2–4 (>20 mm) groups (Table 7).

#### 3.6.1. Associated Factors in the pT1 Group (≤20 mm)

In the univariate analysis for the pT1 group, periA/V (OR 23.897, *p* = 0.001), LI on HE (OR: 4.317, *p* = 0.003), and LI on D2-40 (OR: 2.88, *p* = 0.028) were significant factors. In the multivariate model including these variables, only periA/V (OR 16.08, 95% CI: 2.02–2081.93, *p* = 0.00379) remained independently associated with LNM. Conversely, conventional LI markers lost statistical significance when analyzed alongside periA/V (*p* = 0.11 for HE and *p* = 0.571 for D2-40).

#### 3.6.2. Associated Factors in the pT2–4 Group (>20 mm)

In the pT2–4 group, periA/V was not a significant factor in either the univariate or multivariate analyses (multivariate *p* = 0.378). Conventional LI markers (HE and D2-40) also showed no significant associations. The only feature that remained independently associated with LNM in the multivariate model for this group was retraction artifact (OR: 2.80, 95% CI: 1.01–7.75, *p* = 0.0469).

### 3.7. Subgroup Analysis Within pT1 Categories

To further examine the relationship between invasive tumor size and histological indicators among pT1 tumors, pT1 cases (*n* = 130) were subdivided into pT1a (≤5 mm; *n* = 11), pT1b (6–10 mm; *n* = 44), and pT1c (11–20 mm; *n* = 75) based on invasive size (Table 8).

#### 3.7.1. pT1a Group (≤5 mm)

No LNM was observed in this group (0/11). periA/V was present in 27.3% (3/11) of the cases, and LI on both HE and D2-40 staining was observed in 9.1% (1/11).

#### 3.7.2. pT1b Group (6–10 mm)

Five cases of LNM were identified. periA/V was present in all node-positive cases (100.0%, 5/5). In the node-negative group (*n* = 39), periA/V was present in 82.1% of cases (*p* = 0.574). The sensitivity of conventional LI markers in node-positive cases was low (HE: 40.0% [2/5]; D2-40: 20.0% [1/5]).

#### 3.7.3. pT1c Group (11–20 mm)

Twenty-six cases of LNM were identified. periA/V was present in all node-positive cases (100.0%, 26/26), showing a significant difference compared with node-negative cases (75.5%, 37/49; *p* = 0.006). In contrast, LI sensitivity remained low (34.6% [9/26] for both HE and D2-40).

Overall, among node-positive pT1 tumors with invasive size ≥6 mm (pT1b–c), periA/V was consistently present.

### 3.8. Logistic Regression Analysis by pT1 Subcategories

To further evaluate the factors associated with LNM among pT1 tumors, we performed separate logistic regression analyses for the pT1b (6–10 mm) and pT1c (11–20 mm) subgroups (Table 9).

#### 3.8.1. Logistic Regression in pT1b Group (6–10 mm)

In the pT1b group, no histological indicators were significantly associated with LNM in either the univariate or multivariate analyses.

#### 3.8.2. Logistic Regression in pT1c Group (11–20 mm)

In the univariate analysis for the pT1c group, periA/V (OR 17.667, *p* = 0.003) and LI on HE (OR: 3.076, *p* = 0.045) were significantly associated with LNM. In multivariate analysis, only periA/V (OR: 14.7, 95% CI: 1.687–1935.315, *p* = 0.01) remained independently associated with LNM.

## 4. Discussion

In this study, we demonstrated that perivascular invasion (periA/V) identified on EVG-stained sections is a high-sensitivity histological surrogate marker associated with LNM in IBC-NST. Although we previously reported periA/V as an independent, highly sensitive indicator of LNM in IBC-NST [1], the current analysis further showed that its association with LNM is strongly influenced by the invasive tumor size. In particular, among pT1 tumors, periA/V showed superior sensitivity and NPV compared with conventional LI indicators. Notably, periA/V was present in all node-positive pT1 cases. These findings suggest that, in small tumors, periA/V negativity may potentially be associated with node-negative status, which is often underestimated by conventional pathological assessments.

The remarkably high sensitivity and NPV observed in this study may be explained by the anatomical characteristics of breast tissue. Peripheral lymphatic vessels commonly run in close proximity to arteries and veins, and perivascular connective tissue constitutes an important pathway for lymphatic drainage [1,27,28,29]. PeriA/V identified on EVG-stained sections may sensitively capture tumor spread into, or immediate proximity to, the adventitial lymphatic network. In contrast, conventional LI assessment on HE-stained sections or by D2-40 immunostaining generally requires recognition of tumor cells within an identifiable lymphatic space; however, these delicate luminal structures can be collapsed or disrupted by tumor infiltration, leading to underdetection during routine histopathological evaluation. Using elastic fibers in the vascular wall as stable histological landmarks, periA/V enables consistent identification of the perivascular microanatomical compartment even when the lymphatic vessels themselves are difficult to visualize. Consequently, periA/V may reflect lymphatic invasion with a higher sensitivity than conventional LI markers by capturing tumor involvement along established lymphatic drainage pathways.

LI is generally considered to biologically precede LNM and is therefore expected to be observed more frequently at pathological diagnosis. However, some studies have reported LNM without detectable LI on conventional assessment, suggesting that traditional methods may underestimate true LI. PeriA/V was developed to address this limitation by focusing on the perivascular region, where lymphatic vessels are concentrated in breast tissue, and by using EVG-stained sections to visualize perivascular elastic fibers as reliable landmarks. This approach captures LI as a surrogate based on direct contact between tumor nests and perivascular elastic fibers. Consequently, this design often results in low specificity and a low positive likelihood ratio. PeriA/V is intended to detect LI as an earlier event in the metastatic cascade, rather than to confirm that nodal metastasis has already occurred. PeriA/V positivity in node-negative cases may reflect true LI that has not yet progressed to overt nodal metastasis, rather than representing false positivity.

Conversely, the observation that the association between periA/V and LNM diminished with increasing tumor size—particularly in pT2–4 cases—is an important finding. In a simplified geometric model, doubling the tumor diameter results in a fourfold increase in cross-sectional area and an eightfold increase in volume (V ∝ r^3^). As tumor burden increases, the tumor involvement of the surrounding stroma and perivascular microenvironment becomes more extensive. Given its inherently high sensitivity, periA/V may become a common morphological finding in larger tumors, irrespective of nodal status. In other words, periA/V appears to have the greatest discriminatory value before this “ceiling effect” is reached: it functions as a high-sensitivity indicator associated with LNM in small tumors, whereas in larger tumors, the finding becomes ubiquitous, reducing its ability to distinguish nodal status. In pT1c tumors, periA/V was present in all node-positive cases and showed a statistically significant association with LNM, with periA/V negativity confined to node-negative cases. A similar pattern was observed in pT1b tumors; however, statistical significance was not reached, which likely reflects the limited sample size. Notably, no periA/V-negative cases with LNM were identified in either subgroup. These observations suggest that periA/V negativity may be associated with node-negative status in pT1 tumors.

Furthermore, when periA/V was assessed alongside key clinicopathological variables—including invasive tumor size, nuclear grade, histological grade, multifocality, and conventional LI markers—it remained an independent predictor of LNM (OR 12.4, 95% CI 2.59–59.7, *p* = 0.002). Notably, LI on HE and LI on D2-40 were not independently associated with LNM in this model, whereas invasive tumor size (OR 3.73, *p* < 0.001) and multifocality (OR 2.88, *p* = 0.004) were. These findings suggest that periA/V and clinicopathological risk factors may capture distinct dimensions of LNM association in this cohort: clinicopathological factors such as tumor size and multifocality were associated with increased LNM risk, whereas periA/V showed a stronger association with LNM through its negativity rather than its positivity.

The periA/V assessment on EVG-stained sections differs methodologically from conventional LI evaluation using D2-40 immunohistochemistry, which can be limited by longer turnaround times and higher costs. In addition, interpretive variability may arise because D2-40 can stain the mammary myoepithelium and other non-lymphatic structures, and lymphatic spaces may be obscured or altered by tumor infiltration and stromal reactions [20,21]. In contrast, EVG staining is relatively inexpensive and widely available in most pathology laboratories [30,31]. Importantly, EVG staining provides clear and specific visualization of elastic fibers as stable histological landmarks. Elastic fibers in the vascular wall are sharply highlighted in dark purple to black, making periA/V assessment less dependent on subjective interpretation and potentially more reproducible. The clarity of EVG-stained sections may also facilitate communication between pathologists and surgeons, who have long been troubled by the discrepancy between confirmed LNM and the absence of detectable LI on conventional stains. Although EVG staining has traditionally been used to evaluate VI, the present findings suggest that EVG-based periA/V may reflect lymphatic involvement through histological proximity to perivascular structures.

In this study, periA/V was evaluated in surgical specimens. Its morphological basis may facilitate pathologist–clinician communication by providing a potential explanation for the diagnostic discrepancy between LNM-positive status and the absence of detectable LI on conventional assessment. This communicative value is underpinned by the morphological and anatomical nature of the finding: pathologists can visualize tumor nests in direct contact with perivascular elastic fibers and conceptually reconstruct the process of lymphatic invasion—even in the absence of overt tumor cells within lymphatic lumina—thus providing biological plausibility to periA/V as a surrogate for lymphatic involvement. Whether periA/V assessment could be extended to core needle biopsy specimens remains to be explored in future studies.

This study has some limitations. First, it was a retrospective, single-center study involving 213 patients treated between 2018 and 2023, without any external or internal validation. The wide confidence intervals observed in certain regression analyses reflect near-complete separation, and although Firth’s correction was applied, potential model instability and overfitting cannot be entirely excluded. Therefore, the reported diagnostic performance metrics should be interpreted with caution, as they may overestimate true performance. Multicenter prospective studies with independent validation cohorts are warranted to assess the generalizability of these findings. Second, the pathological evaluation was conducted without blinding and performed using a consensus-based approach involving multiple pathologists. Although this may introduce observer bias, collaborative review of diagnostically challenging cases is common in routine clinical pathology. Thus, our approach reflects real-world diagnostic workflows. The use of elastic fibers as stable histological landmarks on EVG-stained sections is expected to reduce subjective interpretation and enhance reproducibility compared with conventional LI assessment. Third, the sample sizes within the pT1 subcategories (pT1a, pT1b, and pT1c) were limited, making the subgroup logistic regression analyses exploratory. Therefore, the wide confidence intervals preclude drawing definitive conclusions. These findings warrant cautious interpretation and require validation in larger prospective cohorts. Fourth, to maintain cohort homogeneity, we focused solely on IBC-NST; therefore, the diagnostic value of periA/V in other histological types remains unverified. Additionally, the diagnostic value of periA/V relative to other emerging lymphatic invasion markers, such as LYVE-1 and VEGFR-3, has yet to be established, as periA/V represents a conceptually distinct approach based on perivascular contact rather than assessing intraluminal invasion. Fifth, we used simplified criteria for vascular classification, distinguishing arteries from veins primarily based on the presence of an internal elastic lamina on EVG-stained sections. Although this practical approach may improve reproducibility and consistency in routine diagnosis, it does not provide a strict histological categorization of all vessel types. Future studies incorporating additional vascular markers may provide further insights into the nature of perivascular tumor spread.

## 5. Conclusions

PeriA/V, a histological parameter defined by direct tumor contact with perivascular elastic fibers on EVG-stained sections, may reflect LI through histological proximity to perivascular structures. This surrogate indicator was identified as a high-sensitivity LNM-associated indicator and an independent predictor in this cohort, with a particularly strong association with LNM observed in pT1 IBC-NST. Its diagnostic value in this cohort resided primarily in its negativity rather than its positivity. Notably, periA/V negativity in pT1 IBC-NST was consistently observed among node-negative cases in this cohort. These findings, obtained from a single-center retrospective cohort, suggest that periA/V may contribute to LNM risk assessment in early-stage breast cancer.

## Figures and Tables

**Table 1:** Detailed patients’ clinicopathological characteristics.

Characteristic	Patients (*n* = 213)	Characteristic	Patients (*n* = 213)	Characteristic	Patients (*n* = 213)
Age—yrs		Invasive tumor size (greatest dimension, mm)		ER (J-score)	
Average	60.7	Average	19.8	3b	193
Median	61	Median	17	3a	6
Range	23–92	Range	2–90	2	1
premenopausal	72	Total tumor size (mm)		1	2
postmenopausal	141	Average	37.9	0	11
Sex		Median	28	PgR (J-score)	
Male	0	Range	5–135	3b	130
Female	213	Pathological T categories		3a	51
Operative method		1 (up to 20 mm)	130	2	10
Mastectomy	107	1a (1–5 mm)	11	1	1
Partial mastectomy	106	1b (6–10 mm)	44	0	21
Lymph node dissection		1c (11–20 mm)	75	HER2 score	
performed	52	2 (21–50 mm)	73	3+	10
not performed	161	3 (>50 mm)	6	2+ and FISH(+)	8
primary tumor location		4	4	2+ and FISH(−)	27
Left breast	106	Regional lymph node metastasis		1+	130
Right breast	107	N0	126	0	38
Anatomical Subsites		N+ (including N0(i+), N1mi, N1-3)	87	Ki-67 labeling index (hot spot)	
Upper-inner quadrant	51	N0(i+)	7	Average (%)	25.9
Lower-inner quadrant	28	N1mi	14	Median (%)	21.1
Upper-outer quadrant	90	N1	46	Range (%)	0.5–88.6
Lower-outer quadrant	27	N2	14	>20%	113
Central portion	12	N3	6	≤20%	100
Axillary tail	5	Distant metastasis			
Nuclear grade		M0	211		
1	87	M1	2		
2	37	pTNM stage			
3	89	IA	104		
Histological grade		IB	5		
I	73	IIA	45		
II	84	IIB	32		
III	56	IIIA	17		
Unifocal or multifocal		IIIB	3		
Unifocal	140	IIIC	5		
multifocal	73	IV	2		

ER, estrogen receptor; PgR, progesterone receptor; HER2, Human Epidermal Growth Factor Receptor 2; FISH, Fluorescence in situ hybridization.

**Table 2:** Detailed correlations between the lymph node metastasis and clinicopathological variables.

	N0 (*n* = 126) Number (%)	N0(i+) and N1-3 (*n* = 87) Number (%)	*p*-Value		N0 (*n* = 126) Number (%)	N0(i+) and N1-3 (*n* = 87) Number (%)	*p*-Value
Age				PeriA			
Premenopausal	39 (31.0)	33 (37.9)	0.306	(+)	68 (54.0)	60 (69.0)	**0.033**
Postmenopausal	87 (69.0)	54 (62.1)		(−)	58 (46.0)	27 (31.0)	
Primary tumor location				PeriV			
Left breast	66 (52.4)	40 (46.0)	0.404	(+)	90 (71.4)	83 (95.4)	**<0.001**
Right breast	60 (47.6)	47 (54.0)		(−)	36 (28.6)	4 (4.6)	
Anatomical Subsites				PeriA/V			
Upper-inner quadrant	33 (26.2)	18 (20.7)	0.417	(+)	97 (77.0)	85 (97.7)	**<0.001**
Lower-inner quadrant	13 (10.3)	15 (17.2)		(−)	29 (23.0)	2 (2.3)	
Upper-outer quadrant	56 (44.4)	34 (39.1)		PeriD			
Lower-outer quadrant	13 (10.3)	14 (16.1)		(+)	93 (73.8)	74 (85.1)	0.062
Central portion	7 (5.6)	5 (5.7)		(−)	33 (26.2)	13 (14.9)	
Axillary tail	4 (3.2)	1 (1.1)		Lymphatic invasion in HE staining			
Nuclear grade				(+)	20 (15.9)	29 (33.3)	**0.005**
1	67 (53.2)	20 (23.0)	**<0.001**	(−)	106 (84.1)	58 (66.7)	
2	20 (15.9)	17 (19.5)		Lymphatic invasion in D2-40 staining			
3	39 (31.0)	50 (57.5)		(+)	23 (18.3)	31 (35.6)	**0.006**
Histological grade				(−)	103 (81.7)	56 (64.4)	
I	56 (44.4)	17 (19.5)	**<0.001**	Venous invasion in HE staining			
II	45 (35.7)	39 (44.8)		(+)	9 (7.1)	18 (20.7)	**0.006**
III	25 (19.8)	31 (35.6)		(−)	117 (92.9)	69 (79.3)	
Unifocal or Multifocal				Venous invasion in EVG staining			
Unifocal	93 (73.8)	47 (54.0)	**0.003**	(+)	49 (38.9)	66 (75.9)	**<0.001**
Multifocal	33 (26.2)	40 (46.0)		(−)	77 (61.1)	21 (24.1)	
Invasive tumor size (mm)				Retraction artifact			
>20 mm	28 (22.2)	56 (64.4)	**<0.001**	(+)	44 (34.9)	51 (58.6)	**<0.001**
≤20 mm	98 (77.8)	31 (35.6)		(−)	82 (65.1)	36 (41.4)	
				Perineural Invasion			
				(+)	29 (23.0)	31 (35.6)	0.062
				(−)	97 (77.0)	56 (64.4)	

PeriA, Periarterial invasion; PeriV, Perivenous invasion; PeriA/V, Periarterial or perivenous invasion; PeriD, Periductal invasion; EVG, Elastica–van Gieson; HE, hematoxylin and eosin. Bold values indicate statistically significant results.

**Table 3:** Contingency table method.

	Sensitivity	Specificity	PPV	NPV	LR+	LR−
PeriA	69.00% (58.1–78.5%)	46.00% (37.1–55.1%)	46.90% (38.0–55.9%)	68.20% (57.2–77.9%)	1.28 (1.03–1.58)	0.67 (0.47–0.97)
PeriV	95.40% (88.6–98.7%)	28.60% (20.9–37.3%)	48.00% (40.3–55.7%)	90.00% (76.3–97.2%)	1.34 (1.18–1.51)	0.16 (0.06–0.44)
PeriA/V	97.70% (91.9–99.7%)	23.00% (16.0–31.4%)	46.70% (39.3–54.2%)	93.50% (78.6–99.2%)	1.27 (1.15–1.40)	0.1 (0.02–0.41)
PeriD	85.10% (75.8–91.8%)	26.20% (18.8–34.8%)	44.30% (36.6–52.2%)	71.70% (56.5–84.0%)	1.15 (1.01–1.32)	0.57 (0.32–1.02)
Lymphatic invasion in HE staining	33.30% (23.6–44.3%)	84.10% (76.6–90.0%)	59.20% (44.2–73.0%)	64.60% (56.8–71.9%)	2.1 (1.27–3.46)	0.79 (0.67–0.94)
Lymphatic invasion in D2-40 staining	35.60% (25.7–46.6%)	81.70% (73.9–88.1%)	57.40% (43.2–70.8%)	64.80% (56.8–72.2%)	1.95 (1.23–3.11)	0.79 (0.66–0.94)
Venous invasion in HE staining	20.70% (12.8–30.7%)	92.90% (86.9–96.7%)	66.70% (46.0–83.5%)	62.90% (55.5–69.9%)	2.9 (1.37–6.14)	0.85 (0.76–0.96)
Venous invasion in EVG staining	75.90% (65.5–84.4%)	61.10% (52.0–69.7%)	57.40% (47.8–66.6%)	78.60% (69.1–86.2%)	1.95 (1.52–2.50)	0.39 (0.27–0.59)
Retraction artifact	58.60% (47.6–69.1%)	65.10% (56.1–73.4%)	53.70% (43.2–64.0%)	69.50% (60.3–77.6%)	1.68 (1.25–2.26)	0.64 (0.48–0.84)
Perineural Invasion	35.60% (25.6–46.6%)	77.00% (68.6–84.0%)	51.70% (38.4–64.8%)	63.40% (55.2–71.0%)	1.55 (1.01–2.37)	0.84 (0.70–1.00)

PeriA, Periarterial invasion; PeriV, Perivenous invasion; PeriA/V, Periarterial or perivenous invasion; PeriD, Periductal invasion; EVG, Elastica–van Gieson; HE, hematoxylin and eosin; PPV, positive predictive value; NPV, negative predictive value; LR+, positive likelihood ratio; LR-, Negative likelihood ratio; CI, confidence interval; values in parentheses indicate 95% CIs.

**Table 4:** Univariate and multivariate analyses of potential predictors of lymph node metastasis in 213 patients with IBC-NST.

	Univariate			Multivariate			
	Odds Ratio	95% CI	*p*-Value	Odds Ratio	95% CI	*p*-Value	VIF
PeriA	1.900	1.070–3.360	**0.029**				
PeriV	8.300	2.830–24.30	**<0.001**				
PeriA/V	12.700	2.940–54.80	**0.001**	9.690	2.160–43.50	**0.003**	1.031
PeriD	2.020	0.992–4.110	0.053				
Lymphatic invasion with HE stain	2.650	1.380–5.090	**0.003**	1.330	0.572–3.110	0.505	1.540
Lymphatic invasion with D2-40	2.480	1.320–4.650	**0.005**	1.500	0.659–3.400	0.335	1.528
Retraction artifact	2.640	1.500–4.63	**0.001**	2.410	1.300–4.480	**0.005**	1.077
Perineural Invasion	1.850	1.010–3.390	**0.046**	1.570	0.820–3.020	0.173	1.048

PeriA, Periarterial invasion; PeriV, Perivenous invasion; PeriA/V, Periarterial or perivenous invasion; PeriD, Periductal invasion; EVG, Elastica–van Gieson; HE, hematoxylin and eosin; CI, confidence interval; VIF, variance inflation factor. Bold values indicate statistically significant results.

**Table 5:** Independent predictive value of periA/V after adjustment for clinicopathological variables: univariate and multivariate logistic regression in 213 patients with IBC-NST.

	Univariate			Multivariate			
	Odds Ratio	95% CI	*p*-Value	Odds Ratio	95% CI	*p*-Value	VIF
PeriA/V	12.700	2.940–54.80	**0.001**	12.400	2.590–59.70	**0.002**	1.055
Nuclear grade (1 vs. 2–3)	3.800	2.070–7.000	**<0.001**	1.800	0.765–4.230	0.179	1.566
Histological grade (1 vs. 2–3)	3.290	1.740–6.220	**<0.001**	1.660	0.679–4.030	0.267	1.551
Multifocality (Unifocal or Multifocal)	2.400	1.340–4.280	**0.003**	2.880	1.420–5.850	**0.004**	1.065
Invasive tumor size (pT1 vs. pT2-4)	6.320	3.440–11.60	**<0.001**	3.730	1.860–7.460	**<0.001**	1.118
Lymphatic invasion with HE stain	2.650	1.380–5.090	**0.003**	1.170	0.447–3.050	0.752	1.637
Lymphatic invasion with D2-40	2.480	1.320–4.650	**0.005**	1.370	0.540–3.470	0.507	1.623

Hematoxylin and eosin (HE), confidence interval (CI), variance inflation factor (VIF). Bold values indicate statistically significant results.

**Table 6:** Stratified analysis by tumor size (pT1 vs. pT2-4).

pT1				pT2-4			
	N0 (*n* = 99) Number (%)	N0(i+) and N1-3 (*n* = 31) Number (%)	*p*-Value		N0 (*n* = 27) Number (%)	N0(i+) and N1-3 (*n* = 56) Number (%)	*p*-Value
PeriA/V				PeriA/V			
(+)	72 (72.7)	31 (100.0)	**<0.001**	(+)	25 (92.6)	54 (96.4)	0.593
(−)	27 (27.3)	0 (0.0)		(−)	2 (7.4)	2 (3.6)	
Lymphatic invasion in HE staining				Lymphatic invasion in HE staining			
(+)	11 (11.1)	11 (35.5)	**0.004**	(+)	9 (33.3)	18 (32.1)	1.000
(−)	88 (88.9)	20 (64.5)		(−)	18 (66.7)	38 (67.9)	
Lymphatic invasion in D2-40 staining				Lymphatic invasion in D2-40 staining			
(+)	14 (14.1)	10 (32.3)	**0.033**	(+)	9 (33.3)	21 (37.5)	0.810
(−)	85 (85.9)	21 (67.7)		(−)	18 (66.7)	35 (62.5)	
Retraction artifact				Retraction artifact			
(+)	31 (31.3)	12 (38.7)	0.513	(+)	13 (48.1)	39 (69.6)	0.089
(−)	68 (68.7)	19 (61.3)		(−)	14 (51.9)	17 (30.4)	
Perineural Invasion				Perineural Invasion			
(+)	19 (19.2)	11 (35.5)	0.086	(+)	10 (37.0)	20 (35.7)	1.000
(−)	80 (80.8)	20 (64.5)		(−)	17 (63.0)	36 (64.3)	

PeriA/V, Periarterial or perivenous invasion; EVG, Elastica–van Gieson; HE, hematoxylin and eosin. Bold values indicate statistically significant results.

**Table 7:** Stratified logistic regression analysis by tumor size.

	pT1						
	Univariate			Multivariate			
	Odds Ratio	95% CI	*p*-Value	Odds Ratio	95% CI	*p*-Value	VIF
PeriA/V	23.897	3.161–3063.945	**<0.001**	16.080	2.019–2081.931	**0.004**	1.000
Lymphatic invasion with HE stain	4.317	1.669–11.297	**0.003**	2.790	0.789–10.229	0.110	1.747
Lymphatic invasion with D2-40	2.880	1.126–7.262	**0.028**	1.452	0.381–5.127	0.571	1.737
Retraction artifact	1.394	0.600–3.166	0.434	1.030	0.398–2.554	0.950	1.092
Perineural Invasion	2.316	0.950–5.541	0.064	1.822	0.718–4.617	0.204	1.043
	**pT2-4**						
	**Univariate**			**Multivariate**			
	**Odds ratio**	**95% CI**	***p*** **-value**	**Odds Ratio**	**95% CI**	***p*** **-value**	**VIF**
PeriA/V	2.160	0.288–16.2	0.454	2.630	0.306–22.60	0.378	1.086
Lymphatic invasion with HE stain	0.947	0.357–2.52	0.914	0.626	0.179–2.19	0.464	1.559
Lymphatic invasion with D2-40	1.200	0.457–3.15	0.711	1.270	0.375–4.31	0.699	1.508
Retraction artifact	2.470	0.960–6.36	0.061	2.800	1.010–7.75	**0.047**	1.137
Perineural Invasion	0.944	0.364–2.45	0.906	1.160	0.412–3.25	0.781	1.111

PeriA/V, Periarterial or perivenous invasion; EVG, Elastica–van Gieson; HE, hematoxylin and eosin; CI, confidence interval; VIF, variance inflation factor. Bold values indicate statistically significant results.

**Table 8:** Subgroup analysis within pT1 categories.

pT1a				pT1b				pT1c			
	N0 (*n* = 11) Number (%)	N0(i+) and N1-3 (*n* = 0) Number (%)	*p*-Value		N0 (*n* = 39) Number (%)	N0(i+) and N1-3 (*n* = 5) Number (%)	*p*-Value		N0 (*n* = 49) Number (%)	N0(i+) and N1-3 (*n* = 26) Number (%)	*p*-Value
PeriA/V				PeriA/V				PeriA/V			
(+)	3 (27.3)	0	-	(+)	32 (82.1)	5 (100)	0.574	(+)	37 (75.5)	26 (100)	0.006
(−)	8 (72.7)	0		(−)	7 (17.9)	0 (0)		(−)	12 (24.5)	0 (0)	
Lymphatic invasion in HE staining				Lymphatic invasion in HE staining				Lymphatic invasion in HE staining			
(+)	1 (9.1)	0	-	(+)	3 (7.7)	2 (40)	0.091	(+)	7 (14.3)	9 (34.6)	0.073
(−)	10 (90.9)	0		(−)	36 (92.3)	3 (60)		(−)	42 (85.7)	17 (65.4)	
Lymphatic invasion in D2-40 staining				Lymphatic invasion in D2-40 staining				Lymphatic invasion in D2-40 staining			
(+)	1 (9.1)	0	-	(+)	4 (10.3)	1 (20)	0.470	(+)	9 (18.4)	9 (34.6)	0.157
(−)	10 (90.9)	0		(−)	35 (89.7)	4 (80)		(−)	40 (81.6)	17 (65.4)	
Retraction artifact				Retraction artifact				Retraction artifact			
(+)	3 (27.3)	0	-	(+)	6 (15.4)	2 (40)	0.219	(+)	22 (44.9)	10 (38.5)	0.632
(−)	8 (72.7)	0		(−)	33 (84.6)	3 (60)		(−)	27 (55.1)	16 (61.5)	
Perineural Invasion				Perineural Invasion				Perineural Invasion			
(+)	0 (0)	0	-	(+)	6 (15.4)	2 (40)	0.219	(+)	13 (26.5)	9 (34.6)	0.595
(−)	11 (100)	0		(−)	33 (84.6)	3 (60)		(−)	36 (73.5)	17 (65.4)	

PeriA/V, Periarterial or perivenous invasion; EVG, Elastica–van Gieson; HE, hematoxylin and eosin.

**Table 9:** Logistic regression analysis by pT1 subcategories.

	**pT1b**						
	**Univariate**			**Multivariate**			
	**Odds Ratio**	**95% CI**	***p*** **-Value**	**Odds Ratio**	**95% CI**	***p*** **-Value**	**VIF**
PeriA/V	2.538	0.240–346.838	0.498	2.408	0.131–489.364	0.604	1.000
Lymphatic invasion with HE stain	7.449	0.970–56.312	0.053	8.695	0.801–130.15	0.074	1.665
Lymphatic invasion with D2-40	2.630	0.227–19.430	0.390	1.889	0.101–29.734	0.647	1.438
Retraction artifact	3.681	0.525–23.396	0.177	7.055	0.798–88.376	0.078	1.470
Perineural Invasion	3.681	0.525–23.396	0.177	2.757	0.328–22.638	0.335	1.127
	**pT1c**						
	**Univariate**			**Multivariate**			
	**Odds Ratio**	**95% CI**	***p*** **-Value**	**Odds Ratio**	**95% CI**	***p*** **-Value**	**VIF**
PeriA/V	17.667	2.153–2300.087	**0.003**	14.700	1.687–1935.315	**0.010**	1.000
Lymphatic invasion with HE stain	3.076	1.023–9.61	**0.045**	2.922	0.568–16.79	0.198	2.179
Lymphatic invasion with D2-40	2.314	0.798–6.784	0.121	1.558	0.305–7.509	0.578	1.974
Retraction artifact	0.778	0.294–2.004	0.604	0.430	0.116–1.359	0.155	1.454
Perineural Invasion	1.468	0.528–4.018	0.457	1.063	0.357–3.142	0.911	1.048

PeriA/V, Periarterial or perivenous invasion; EVG, Elastica–van Gieson; HE, hematoxylin and eosin; CI, confidence interval; VIF, variance inflation factor. Bold values indicate statistically significant results.

## Data Availability

The data presented in this study are available upon request from the corresponding author. The data are not publicly available because of privacy and ethical restrictions, as they contain sensitive patient information from a single-center clinical cohort.

## Abbreviations

The following abbreviations are used in this manuscript:

IBC-NST	Invasive breast carcinoma of no special type
LNM	Lymph node metastasis
periA/V	Periarterial or perivenous invasion
periA	Periarterial invasion
periV	Perivenous invasion
periD	Periductal invasion
LI	Lymphatic invasion
VI	Venous invasion
PNI	Perineural invasion
EVG	Elastica–van Gieson
HE	Hematoxylin and eosin
D2-40	Podoplanin (lymphatic endothelial marker)
DCIS	Ductal carcinoma in situ
ER	Estrogen receptor
PgR	Progesterone receptor
HER2	Human epidermal growth factor receptor 2
FISH	Fluorescence in situ hybridization
SSM	Skin-sparing mastectomy
NSM	Nipple-sparing mastectomy
OR	Odds ratio
CI	Confidence interval
NPV	Negative predictive value
PPV	Positive predictive value
LR+	Positive likelihood ratio
LR−	Negative likelihood ratio
VIF	Variance inflation factor
